# Variability in genes regulating vitamin D metabolism is associated with vitamin D levels in type 2 diabetes

**DOI:** 10.18632/oncotarget.26178

**Published:** 2018-10-09

**Authors:** Laura Bertoccini, Diego Bailetti, SUMMER Study in Diabetes Group, Elena Alessi, Raffaella Buzzetti, Maria Gisella Cavallo, Massimiliano Copetti, Efisio Cossu, Paola D'Angelo, Salvatore De Cosmo, Lazzaro Di Mauro, Frida Leonetti, Susanna Morano, Lelio Morviducci, Nicola Napoli, Sabrina Prudente, Giuseppe Pugliese, Vincenzo Trischitta, Marco Giorgio Baroni

**Affiliations:** ^1^ Department of Experimental Medicine, Sapienza University of Rome, Rome, Italy; ^2^ Unit of Biostatistics, IRCSS Casa Sollievo della Sofferenza, San Giovanni Rotondo, Italy; ^3^ Department of Medical Sciences and Public Health, University of Cagliari, Cagliari, Italy; ^4^ Unit of Diabetology, Sandro Pertini Hospital, Rome, Italy; ^5^ IRCSS Casa Sollievo della Sofferenza, Department of Medicine, San Giovanni Rotondo, Italy; ^6^ Laboratory of Clinical Chemistry, IRCSS Casa Sollievo della Sofferenza, San Giovanni Rotondo, Italy; ^7^ Unit of Diabetology, S. Spirito Hospital – AslRM1, Rome, Italy; ^8^ Campus Biomedico University, Rome, Italy; ^9^ IRCCS Casa Sollievo della Sofferenza, Research Unit of Metabolic and Cardiovascular Diseases, San Giovanni Rotondo, Italy; ^10^ Department of Clinical and Molecular Medicine, Sapienza University of Rome, Rome, Italy

**Keywords:** genetic risk score, DHCR7 (7-dehydrocholesterol reductase), CYP2R1 (Cytochrome P450 Family 2 Subfamily R Member 1), GC (Vitamin D Binding Protein), SUMMER Study in Diabetes

## Abstract

Mortality rate is increased in type 2 diabetes (T2D). Low vitamin D levels are associated with increased mortality risk in T2D. In the general population, genetic variants affecting vitamin D metabolism (*DHCR7* rs12785878, *CYP2R1* rs10741657, *GC* rs4588) have been associated with serum vitamin D. We studied the association of these variants with serum vitamin D in 2163 patients with T2D from the “Sapienza University Mortality and Morbidity Event Rate (SUMMER) study in diabetes”. Measurements of serum vitamin D were centralised. Genotypes were obtained by Eco™ Real-Time PCR. Data were adjusted for gender, age, BMI, HbA1c, T2D therapy and sampling season.

DHCR7 rs12785878 (*p* = 1 x 10–4) and *GC* rs4588 (*p* = 1 x 10–6) but not *CYP2R1* rs10741657 (*p* = 0.31) were significantly associated with vitamin D levels.

One unit of a weighted genotype risk score (GRS) was strongly associated with vitamin D levels (*p* = 1.1 x 10–11) and insufficiency (<30 ng/ml) (OR, 95%CI = 1.28, 1.16–1.41, *p* = 1.1 x 10–7).

In conclusion, DHCR7 rs12785878 and *GC* rs4588, but not CYP2R1 rs10741657, are significantly associated with vitamin D levels. When the 3 variants were considered together as GRS, a strong association with vitamin D levels and vitamin D insufficiency was observed, thus providing robust evidence that genes involved in vitamin D metabolism modulate serum vitamin D in T2D.

## INTRODUCTION

The rate of mortality of patients with T2D doubles that of non-diabetic individuals of similar age [1], thus making diabetes a leading risk factor for all-cause mortality worldwide [2]. Therefore, great efforts are needed to tackle such tremendous burden, including the identification of novel biomarkers and the related pathogenic pathways.

The biologically active form of vitamin D, 1,25-dihydroxyvitamin D3 plays a central role in a wide variety of metabolic pathways. Vitamin D insufficiency, affecting as many as 50% healthy adults in developed countries, has been linked to autoimmune [3, 4], infectious [5], cardiovascular [6], neurodegenerative diseases [7] and cancer [8]. Meta-analyses of observational studies have consistently found that vitamin D deficiency (and insufficiency) is associated with an increased risk of cardiovascular mortality and events (i.e. myocardial infarction, heart failure and stroke) [9–19] as well as risk of T2D [20, 21]. In the specific context of mortality rate in T2D, it is worth noticing that low vitamin D levels in diabetic patients have been associated with a 2 fold increased risk of all-cause and CVD mortality [22].

Although serum levels of vitamin D are to some extent under the control of modifiable determinants such as dietary intake and synthesis in the skin, classical twin studies showed that Vitamin D levels are 50–80% heritable [23, 24], thus implying a central role for genetic determinants.

Genes affecting vitamin D metabolism are candidates for the control of serum vitamin D levels.

In this respect, three large GWA studies of serum 25-hydroxyvitamin D reported that variants at three loci reached genome-wide significance above all the others [25–27]. They were rs12785878 in DHCR7, rs10741657 in CYP2R1, rs4588 in GC.

*DHCR7,* which encodes the enzyme 7-dehydrocholesterol reductase, thereby affecting Vitamin D synthesis [28]; *CYP2R1*, which encodes a hepatic microsomal enzyme responsible for vitamin D 25-hydroxylation [29]; and *GC*, which encodes for a multifunctional serum glycoprotein that binds and transports vitamin D and its metabolites [30].

All genetic association between these three genes and Vitamin D levels were observed in the general population. Whether these associations are also observed among patients with T2D is not know. This lack of knowledge is not trivial, given that diabetes per se affects vitamin D levels and that vitamin D insufficiency increases the rate of mortality in T2D [22]. We tried to give our contribution to this subject, by investigating the role of variability at *DHCR7*, *CYP2R1* and *GC* genes, considered either individually or in combination, on serum vitamin D concentrations in a large and very homogeneous cohort of Italian patients with T2D.

## RESULTS

The clinical features of all 2163 patients with T2D are shown in Table 1. On average, patients in the SUMMER study cohort show a mean duration of disease of 11.4 years, an acceptable glucose control (HbA1c 7.3%), and a mean vitamin D level of 23.1 which resulted below sufficiency (i.e. < 30 ng/ml).

**Table 1:** Clinical and biochemical parameters of study population

**Sex (M/F)**	1302/861	
**Age (years)**	66.2 ± 9.8	28–94
**Weight (kg)**	82.9 ± 17.0	39–189
**BMI (kg/m^2^)**	29.9 ± 5.4	15–74
**Waist (cm)**	103.8 ± 13.4	52–230
**HbA1c (%/mmol/mol)**	7.3 ± 1.5/56.0 ± 15.9	4–15/20–142
**T2D duration (years)**	11.4 ± 8.8	0–54
**TC (mg/dl)**	172.2 ± 37.3	69–359
**HDL-C (mg/dl)**	46.7 ± 12.5	17–104
**LDL-C (mg/dl)**	97.2 ± 32.4	8–230
**TG (mg/dl)**	143.4 ± 93.8	30–1576
**SBP (mm/Hg)**	135.4 ± 15.2	90–200
**DBP (mm/Hg)**	79.2 ± 9.0	50–130
**Vitamina D (ng/ml)**	23.1 ± 10.0	7–78
**Anti-hypertension drugs yes/no (%)**	76/24	
**Anti-dyslipidemic drugs yes/no (%)**	63/37	
**Anti hyperglycaemic drugs yes/no (%)**	91.5/8.5	
**- On insulin yes/no (%)**	28/72	

Values are expressed as means ± standard deviations or rate of subjects, as appropriate in the left column and as ranges in the right column. Abbreviations: BMI, body mass index; HbA1c glycosylated hemoglobin SBP, systolic blood pressure; DBP, diastolic blood pressure; TC, total cholesterol; TG, triglycerides; HDL-C, high density lipoprotein-cholesterol; LDL-C, low density lipoprotein-cholesterol.

In all study subjects, genotypes for *DHCR7* rs12785878 T>G (intronic), *CYP2R1* rs10741657 G>A (5′ UTR) and *GC* rs4588 G>T (missense Lys214Glu) variants as well as serum vitamin D levels were determined. We tested the association between DHCR7, CYP2R1 and GC genotypes and clinical characteristics of study participants including gender, age, weight, BMI, waist, HbA1c, T2D duration, blood pressure, total cholesterol, HDL and LDL-cholesterol, and circulating triglycerides. With the exception of age in DHCR7 rs12785878 genotypes (being lower in GG individuals), no significant difference in any clinical variables was observed across genotypes of any SNP (data not shown).

### *DHCR7* rs12785878 T>G

The rs12785878 SNP at *DHCR7* was significantly associated with means (95% CIs) vitamin D levels [means: 23.8 (95%CI = 23.2–24.4), 22.6 (95%CI = 22.0–23.3), 21.1 (95%CI = 19.9–22.4) ng/ml in TT, TG and GG individuals, respectively, *p* = 3.8 × 10^–5^] (Table 2).

**Table 2:** Vitamin D levels of all participants across *DHCR7*, C*YP2R1* and *GC* genotypes

	Genotypes	Vitamin D (ng/ml)
**DHCR7 rs12785878 T>G**	**TT (*n* = 1099)**	23.8 (23.2–24.4)
	**TG (*n* = 854)**	22.6 (22.0–23.3)
	**GG (*n* = 210)**	21.1 (19.9–22.4)
	**Beta-value**	−0.057 (0.014)
	***p*-value**	3.8 × 10^–5^
	adjusted beta-value^*^	−0.058 (0.014)
	adjusted *p*-value^*^	1 × 10^–4^
**CYP2R1 rs10741657 G>A**	**GG (*n* = 1069)**	22.7 (22.1–23.3)
	**GA (*n* = 894)**	23.2 (22.5–23.9)
	**AA (*n* = 200)**	24.7 (23.1–26.4)
	**Beta-value**	0.023 (0.014)
	***p*-value**	0.11
	adjusted beta-value^*^	0.014 (0.014)
	adjusted *p*-value^*^	0.31
**GC rs4588 G>T**	**GG (*n* = 1140)**	24.0 (23.4–24.6)
	**GT (*n* = 845)**	22.3 (21.6–22.9)
	**TT (*n* = 178)**	20.9 (19.6–22.1)
	**Beta-value**	−0.065 (0.014)
	***p*-value**	5.8 × 10^–6^
	adjusted beta-value^*^	−0.072 (0.014)
	adjusted *p*-value^*^	1 × 10^–6^

Values are expressed as means (95% CIs) and beta values (standard errors). *P* values < 0.05 are considered significant.

^*^Adjusted *p*-values were corrected in multiple linear regression for age, gender, BMI, Hba1c, T2D therapy and seasonality.

The observed association was still significant in a multivariable model comprising adjustments for gender, age, BMI, HbA1c and sampling season (adjusted-*p* = 2.1 × 10^–5^). Similarly, no changes were observed in the above-mentioned association when insulin treatment vs. oral anti-hyperglycaemic drugs was taken into account as a covariate (adjusted-*p* = 1 × 10^–4^), in order to address a possible confounding effect of diabetes treatment.

### CYP2R1 rs10741657 G>A

Though not reaching a formal statistical significance, a tendency toward an association was observed between the CYP2R1 rs10741657 SNP and mean (95% CIs) vitamin D levels [means: 24.7 (95%CI = 23.1–26.4), 23.2 (95%CI = 22.5–23.9), 22.7 (95%CI = 22.1–23.3) ng/ml in AA, AG and GG individuals, respectively, *p* = 0.11 and *p* = 0.31 in the unadjusted and adjusted model (gender, age, BMI, HbA1c, T2D therapy and sampling season), respectively].

### GC rs4588 G>T

The GC rs4588 SNP was significantly associated with means (95% CIs) vitamin D levels both in unadjusted analysis [means: 24.0 (95%CI = 23.4–24.6), 22.3 (95%CI = 21.6–22.9), 20.9 (95%CI = 19.6–22.1) ng/ml in GG, GT and TT individuals, respectively, *p* = 5.8 × 10^–6^] (Table 2) and after the inclusion of the possible confounders (gender, age, BMI, HbA1c, T2D therapy and sampling season) into the model (adjusted-*p* = 1 × 10^–6^).

### GRS

**Figure 1:**
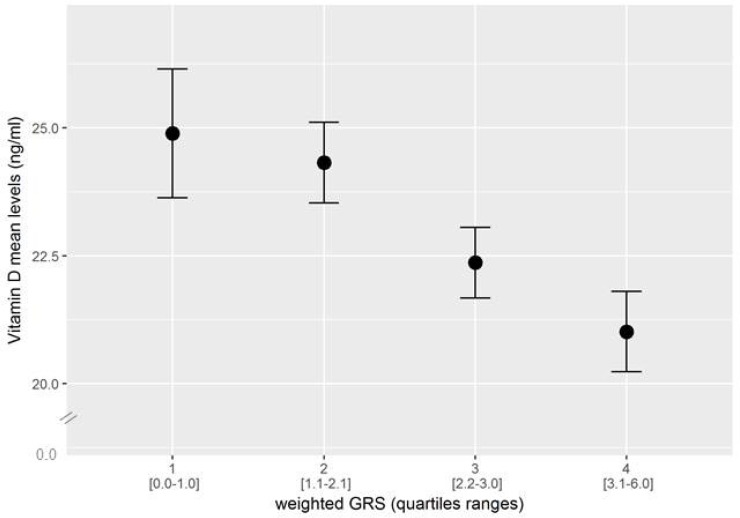
Combined effect of risk alleles, as indicated by quartiles of a weighted genotype risk score (w-GRS), on Vitamin D levels Mean Vitamin D levels significantly decreased as a function of the number of risk alleles (*p* = 1.1 × 10^−7^). In the x-axis, the score range in each quartile is indicated in parenthesis.

Aggregating information from multiple SNPs, each with small effects, into a single genetic risk score (GRS) has become a useful tool for examining the cumulative predictive ability of genetic variation at known loci on different disease outcomes and related phenotypes [31]. To investigate the combined role on vitamin D levels of the three variant considered as a whole, a w-GRS were created, as described in Methods. A strong association was observed between w-GRS and serum vitamin D both in the unadjusted and the adjusted model (beta (SE) = −0.049 (0.008), *p* = 1.6 × 10^–10^, adjusted-*p* = 1.1 × 10^–11^). Similar results were observed when the unw-GRS was used (beta (SE) = −0.049 (0.008); *p* = 2.2 × 10^–9^, adjusted-*p* = 1.3 × 10^–9^); proportions of individuals carrying the different number of risk alleles are shown in Table 3. In addition, vitamin D levels decreased significantly (*p* = 1.6 × 10^–9^) across w-GRS quartiles (Figure 1). Finally, the w-GRS was strongly associated with vitamin D insufficiency, with each score unit increasing the probability (OR) of having vitamin D levels <30 ng/ml by approximately 30% (OR = 1.28, 95%CI, 1.16–1.40, *p* = 2.6 × 10^–7^, adjusted-*p* = 1.1 × 10^–7^).

**Table 3:** Proportion of individuals present in each risk-allele subgroup

risk alleles: *n*	subjects: *n* (%)
0	53 (2.5)
1	317 (14.7)
2	701 (32.4)
3	670 (31)
4	338 (15.6)
5	78 (3.6)
6	6 (0.3)

## DISCUSSION

The present study, carried out in a large population of patients of European ancestry with T2D, is the first testing the association between variability in three genes involved in vitamin D metabolism and serum vitamin D, a biomarker of mortality risk in diabetic patients [22].

We found strong evidence of association between low vitamin D levels and SNPs rs12785878 and rs4588, harboured by *DHCR7* and *GC*, respectively, while only a non-significant trend was observed for rs10741657 at *CYP2R1*. More importantly, when the three SNPs were considered in a combined fashion as indicated by a genetic risk score, a very strong association with both serum vitamin D and hypovitaminosis was observed. Hence, we do have a genetic marker of vitamin D levels, which can be used, for example by a Mendelian randomization approach [32], to address whether the reported association between serum vitamin D and mortality rate [22] in T2D is sustained by a cause-effect relationship.

Our results are consistent with previous studies conducted in the general population, reporting that *DHCR7*, *CYP2R1* and *GC* are key loci involved in the hereditary control of vitamin D levels [25–27, 33]. SNPs in these three genes were therefore chosen because they showed the smallest *p*-values of those reaching genome-wide significance, and have been replicated in a second large study [27], also showing the strongest association between these three genes and vitamin D levels.

Given the known role of T2D itself in lowering vitamin D levels, our present finding clearly indicates that the effect of *DHCR7*, *CYP2R1* and *GC* variability is independent of hyperglycaemia or other intrinsic features of diabetes status. Of note, we observed a mean difference between genotypes of 4–5 ng/dl in vitamin D levels. Although it is reasonable to believe that this difference between genotypes in vitamin D level may not be clinically relevant, it is conceivable that in diabetic subjects, who are already affected by low vitamin D levels, a further genetically induced decrease may have in the long term detrimental consequences. Only longitudinal studies may answer this question.

Strengths of our study are the large, homogeneous and clinically well-characterized cohort of patients with T2D derived from the SUMMER Study in Diabetes [34]. Also, vitamin D levels were determined in all subjects, with a single centralized method. Finally the study was well powered to detect the previously reported association [26] between each genetic variant and serum vitamin D.

We acknowledge that no information on vitamin D supplementation, which might have influenced vitamin D levels in our patients, was available. Epidemiological data from the ARNO Diabetes Italian registry [35] show that only approximately 20% patients with T2D are on vitamin supplementation, also including vitamin D. However, such a treatment should have occurred randomly across genotype groups, thus not interfering with the associations that we report here. Another possible limitation of our study is that, with the exception of GC rs4588 G>T, which results in a Threonine to Lysine amino acid change in codon 436 that determines a protein change from GC-1 to GC-2 with lower affinity for 25(OH)D [36], no functional data are available for the two SNPs in DCHR7 and CYP2R1 genes. However, with respect to CYP2R1 gene, a recent analysis of tagging SNPs in the CYP2R1 locus confirmed the strongest association between Vitamin D levels and rs2060793, a tagging SNP in very strong LD with our rs10741657 [37], which is therefore highly representative of this locus.

In conclusion, this study demonstrates a strong independent association between variants in genes involved in the metabolism of vitamin D and its circulating levels in patients with T2D, establishing for the first time the role of these genetic factors in the regulation of vitamin D levels also in the clinical setting of type 2 diabetes. Based on the present findings, further prospective studies may now be designed aimed at addressing the intrinsic nature of the association between low serum vitamin D and increased mortality rate in T2D [22].

## METHODS

### Study subjects

The first 2163 consecutive study subjects of the “Sapienza University Mortality and Morbidity Event Rate (SUMMER) study in diabetes” cohort were studied. This is an observational, prospective, collaborative study aimed at unravelling new molecular predictors of mortality and vascular morbidity in patients with T2D [34] (Trial registration: ClinicalTrials.gov, NCT02311244; URL: https://clinicaltrials.gov/ct2/show/NCT02311244?termZSUMMER&rankZ5).

Briefly, consecutive patients with T2D of age >18 years and European ancestry were recruited from the outpatient clinics of 10 Italian centres. All patients have undergone a structured interview in order to collect information on family history of diabetes and cardiovascular disease and on current treatments. All subjects have had a complete work-up including clinical examination, anthropometric measurements and laboratory tests. BMI was calculated as body weight (kg)/height (m^2^). The diagnosis of hypertension was based on the presence of elevated systolic (>140 mmHg) and/or diastolic (>90 mmHg) blood pressure, and/or the current use of antihypertensive medications.

### Laboratory determinations

Study populations underwent fasting blood sampling to assess glycosylated hemoglobin (HbA1c), total cholesterol, HDL-cholesterol, triglycerides, white blood cell count, uric acid, serum creatinine and urinary albumin/creatinine ratio (ACR). Low-density lipoprotein (LDL) cholesterol value was obtained using Friedwald formula. In addition, measurements of serum 25-hydroxyvitamin D levels (by chemiluminescent immunoassay from ARUP Laboratory, Salt Lake City, UT, USA) were carried out in all study patients. In order to avoid the introduction of a bias linked to the different inclination of the sunrays during the different seasons of the year, the sampling period was taken into account for the statistical analyses [38].

### Genotyping assay

The following SNPs have been studied in DNAs from all 2163 individuals used for the present study: *DHCR7* rs12785878 T>G, *CYP2R1* rs10741657 G>A, *GC* rs4588 G>T.

Genotyping of SNPs was assayed using the TaqMan assays (Applied Biosystems) C_32063037_10 for *DHCR7* rs12785878 T>G, C_2958430_10 for *CYP2R1* rs10741657 G>A and C_8278879_10 for *GC* rs4588 G>T. The assay was carried out on an Eco^™^ Real-Time PCR System by Illumina (San Diego, CA) in a total volume of 10 μl. After an initial polymerase activation step at 95° C for 3 min, amplification was performed using 45 cycles of denaturation (95° C for 15 s), annealing and extension (60° C for 1 min).

### Statistical analysis

Patients’ baseline characteristics were reported as mean ± SD and percentages for continuous and categorical variables, respectively. Log transformation was used for 25-OH D levels, that was non-normally distributed. Differences between continuous variables across genotype classes were evaluated by ANOVA models. Categorical variables distribution was compared between groups by χ^2^ test. Univariate and multivariate linear regression analysis was used to assess the effect of each single SNP (assuming an additive genetic model of inheritance) on continuous outcome. Multivariable analyses were performed using linear regression models adjusting for gender, age, BMI, HbA1c and sampling season as binary variable (0 = autumn/winter, 1119 subjects and 1 = summer/spring, 1044 subjects). Results were reported as beta values along with their 95% confidence intervals (CIs). A *P*-value < 0.05 was considered as statistically significant. All analyses were performed using SPSS version 20.0 (Chicago, IL, USA).

### Power study

In our study the vitamin D was distributed with standard deviation equal to 10.4. In the whole sample (*n* = 2163), we had at least 96% power with a type I error of 5% and 80% power with a type I error of 1% to detect the same effect size of each SNP described by Wang et al. [26] in the Framingham Heart Study.

### Genetic risk score

An unweighted genotype risk score (unw-GRS) was created by summing the number of the risk alleles associated with lower vitamin D levels of the 3 SNPs, carried by each subject. A weighted genotype risk score (w-GRS) was created by summing the weighted effect size on serum vitamin D of the 3 SNPs carried by each subject.

### Ethics statement

The study was conducted in accordance with the Declaration of Helsinki. The study protocol has been approved by the coordinating centre’s Ethic Committee and, thereafter, by the Ethics Committee of each centre outside the Umberto I “Sapienza” University Hospital, in Rome. Written consent was obtained from all subjects before the study.

## Abbreviations

T2D: type 2 diabetes
BMI: body mass index
HbA1c: glycosylated hemoglobin
SBP: systolic blood pressure
DBP: diastolic blood pressure
TC: total cholesterol
TG: triglycerides
HDL-C: high density lipoprotein-cholesterol
LDL-C: low density lipoprotein-cholesterol
ACR: urinary albumin/creatinine ratio
Cis: confidence intervals
unw-GRS: unweighted genotype risk score
w-GRS: weighted genotype risk score
DHCR7: 7-dehydrocholesterol reductase
CYP2R1: Cytochrome P450 Family 2 Subfamily R Member 1
GC: Vitamin D Binding Protein
